# Mammography correlates to better survival rates in breast cancer patients: a 20-year experience in a University health institution

**DOI:** 10.3332/ecancer.2020.1005

**Published:** 2020-01-23

**Authors:** Cristóbal Maiz, Fernando Silva, Francisco Domínguez, Héctor Galindo, Mauricio Camus, Augusto León, David Oddó, Alejandra Villarroel, Dravna Razmilic, María Elena Navarro, Lidia Medina, Tomás Merino, Eugenio Vines, José Peña, Daniela Maldonado, Mauricio P. Pinto, Francisco Acevedo, César Sánchez

**Affiliations:** 1 Departament of Oncological and Maxillofacial Surgery, School of Medicine, Pontificia Universidad Católica de Chile, 8330032 Santiago, Chile; 2 Departament of Hematology and Oncology, School of Medicine, Pontificia Universidad Católica de Chile, 8330032 Santiago, Chile; 3 Departament of Anatomic Pathology, School of Medicine, Pontificia Universidad Católica de Chile, 8330032 Santiago, Chile; 4 Departament of Radiology, School of Medicine, Pontificia Universidad Católica de Chile, 8330032 Santiago, Chile; 5 Cancer Center ‘Nuestra Señora de la Esperanza’, Pontificia Universidad Católica de Chile, 8330032 Santiago, Chile

**Keywords:** breast neoplasia, prognosis, survival, mammography, advanced breast cancer

## Abstract

Breast cancer (BC) is the most common malignancy in women. We retrieved medical records from >2,000 Chilean BC patients over the 1997–2018 period. The objective was to assess changes in clinical presentation or prognosis of our patients throughout these 20 years of practice. Although most variables did not display significant variations, we observed a progressive increase in stage IV BC over this period. Our data showed that tumour stage III/IV or HER2-enriched subtype tumours were associated with poorer prognosis. In contrast, we found that patients diagnosed by mammography had better overall survival. We speculate that better screenings and more sensitive imaging could explain the unexpected rise in stage IV cases. Our results support mammography screenings as an effective measure to reduce BC-related mortality.

## Introduction

Worldwide, breast cancer (BC) is a high incidence cancer among women. In Chile, it is women’s leading cause of cancer death [1]. Reports indicate an increase in BC incidence [1, 2] in recent decades. Several factors could explain this observation: changes in lifestyle and better screenings along with an aging population.

The widespread use of mammography, better treatments and supportive care have allowed a progressive improvement in prognosis and a sustained 1%–2% increase in survival every year over the last 30 years in developed countries [1, 3].

Evidently, changes in diagnostic methods and staging of the disease [4] may be an interpretation bias in cancers associated to good prognosis (such as breast). This study analysed the clinical features of BC patients diagnosed at our institution over 20 years. Our data indicates a progressive increase in stage IV cases over time. Importantly, we confirmed that the diagnosis by mammography was associated with reduced mortality.

## Methods

### Patients and BC subtypes

Retrospective analysis of patients with invasive BC was performed, including all the women treated between January 1997 and August 2018 at Nuestra Señora de la Esperanza Cancer Centre in the Pontificia Universidad Católica de Chile or the Red de Salud UC-Christus Health Network, that were registered in our database. The Scientific and Ethics Committee approved this study. Assessed variables included: age at diagnosis, reason for attending the clinic (RAC) (this is the reason why patients consulted the clinic), dividing those who were motivated by symptoms or signs and those who were motivated by routine mammography findings, TNM (Tumor, Nodes, Metastases) stage [5], histological grade (HG) and tumour subtype. Mammograms referred as RAC were performed as BC screening. Patients who consulted for symptoms or signs of BC also had mammograms as part of workout, but those were not their reason for consultation. Survival rates were calculated and censored according to last follow-up date. Tumour subtypes were defined as Luminal A/B, Human Epidermal Growth Factor Receptor type-2 (HER2) [6]-enriched or Triple Negative (TN) as described [7].

### Statistical analysis

According to their distribution, data were presented as average ± standard deviation or median (range). Categorical variables are presented as frequency or percentage. Continuous variables were compared by Student’s t-test, categorical variables were compared by chi-square or Fisher’s exact; *p*-value < 0.05 was considered statistically significant. Analyses were performed in SPSS v21 (IBM).

## Results

**Figure 1.**
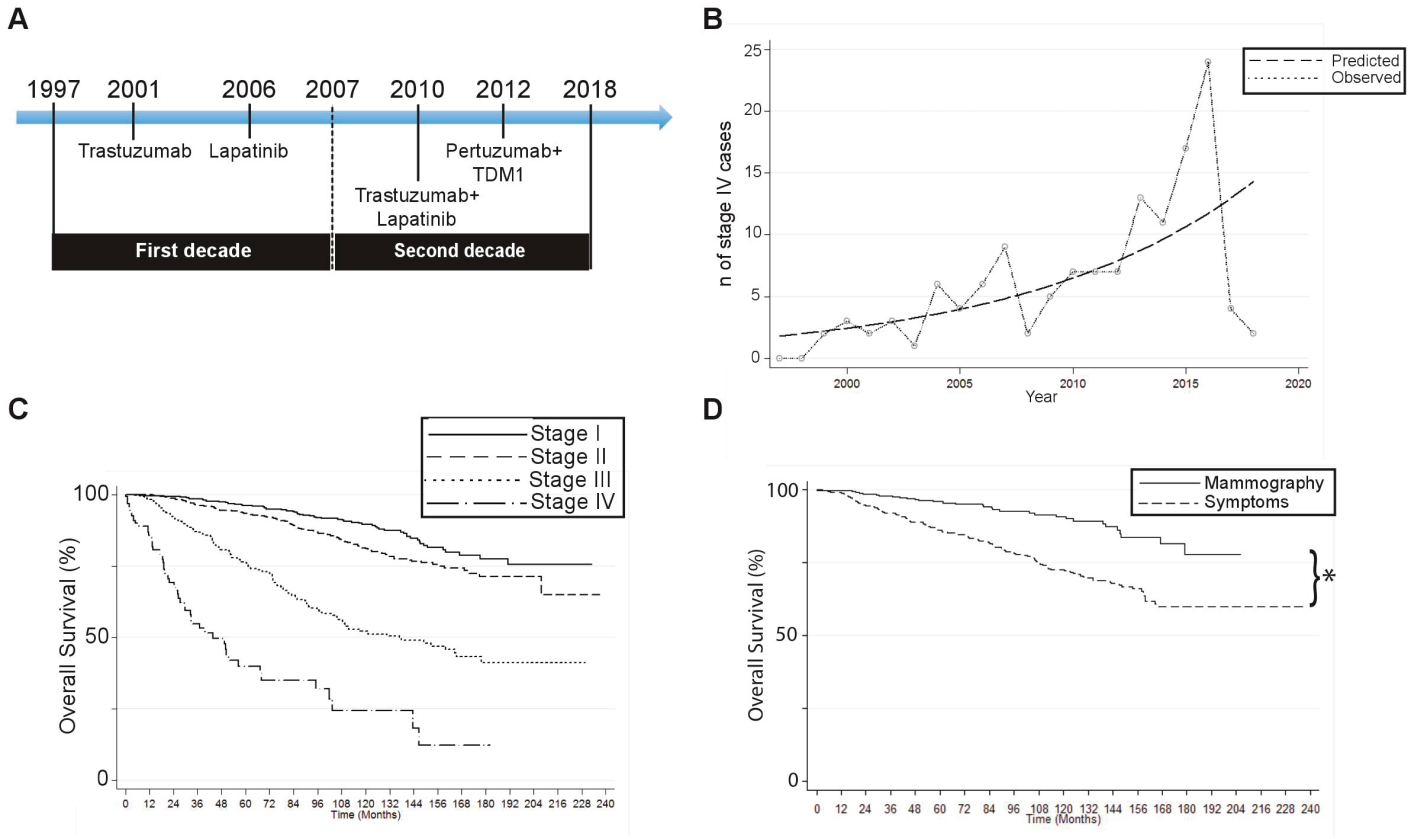
A 20-year experience: main results. (A): Study timeline including year of approval for HER2 BC treatments. (B): Number of stage IV cases over time. Chart compares predicted versus observed cases. (C): Overall survival rates on patients by tumour stage. (D): Overall survival rates on patients diagnosed by screening mammography or by symptoms. (*p < 0.05 by log-rank).

Medical records from 2,723 BC patients diagnosed between 1997 and 2018 were analysed (Figure 1A). Clinical characteristics of patients are summarised in Table 1. Age at diagnosis during the first (1997–2007) or the second decade (2008–2018) were similar (55.4 ± 12.9 versus 55.9 ± 13.2, respectively; *p* = 0.34). Tumour stage at diagnosis was obtained for 2,470 cases. As expected, most patients were stage I/II (74.4% combined). Tumour subtype was obtained for 2,286 patients (84%). Again as expected, the majority were luminal/hormone dependent BCs (Luminal A/B: 81.3%; Table 1). The RAC was obtained on 1,754 patients (64.4%); 36.9% (*n* = 648) were diagnosed by mammography, the remaining 63.1% (*n* = 1,106) by symptoms. Especially, % of stage I was significantly higher among those diagnosed by mammography compared to symptoms (58.55% versus 17%, *p* < 0.0001, Table 2). The proportion of patients diagnosed by mammography in the first versus the second decade were alike (*p* = 0.194).

Interestingly, we found a progressive and significant increase in stage IV cases over the assessed period (Figure 1B). A Poisson regression model demonstrates a 3%/year increase in stage I/II/III patients versus a 11%/year increase in stage IV (*p* = 0.0001). Solid line in Figure 1B represents a linear regression estimate of a 7% cumulative increase in stage IV cases in 20 years (predicted) versus the incidence of stage IV BC cases (observed). HG was obtained on 1,284 cases (47.2%): 17.9% were HG1 (*n* = 230), 40% GH2 (*n* = 514), and 42.1% HG3 (*n* = 540).

## Overall survival

**Figure S1.**
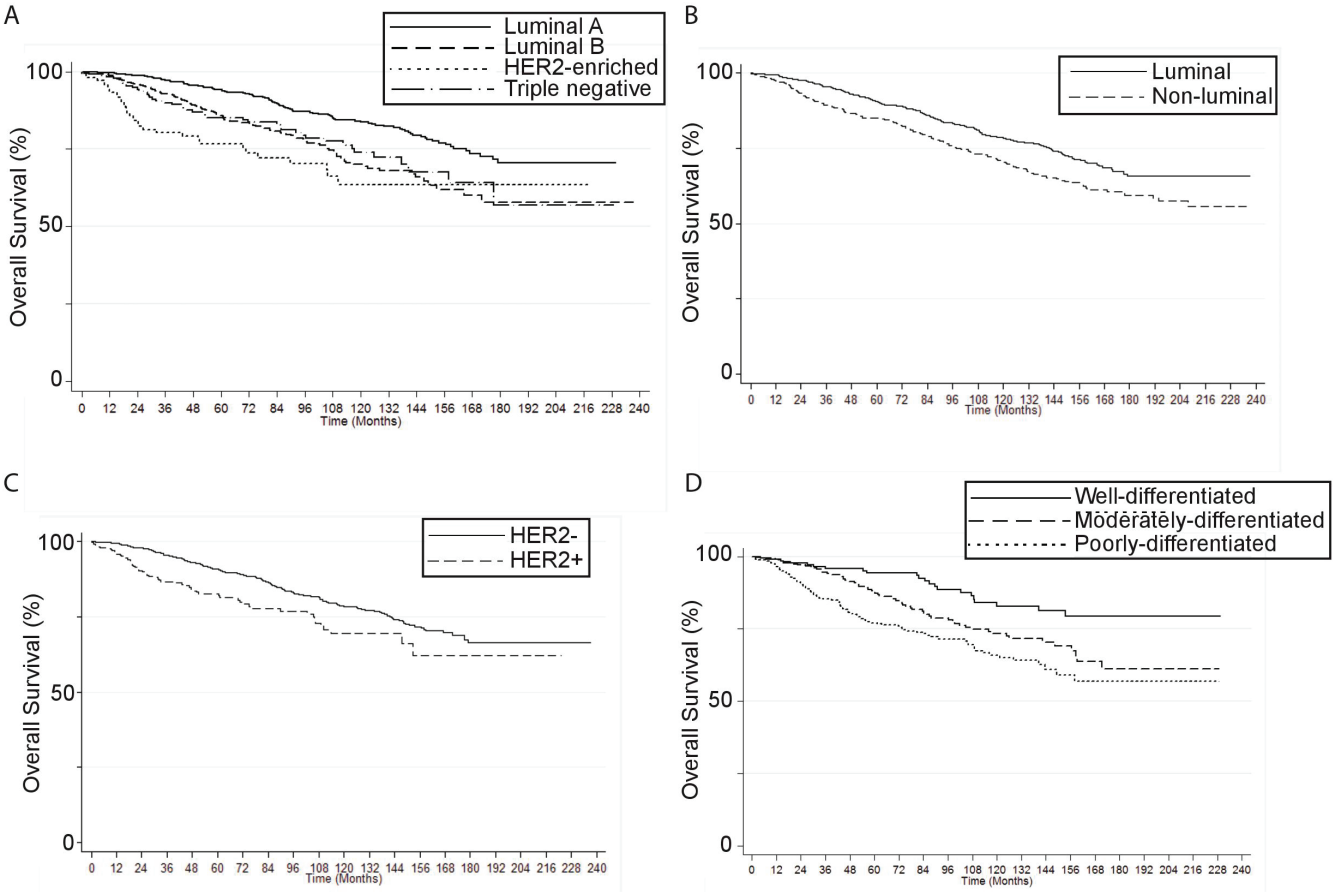
Breast cancer overall survival by (A) molecular subtype, (B) luminal vs non-luminal, (C) HER2 status, and (D) histological grade (Kaplan Meier).

**Figure S2.**
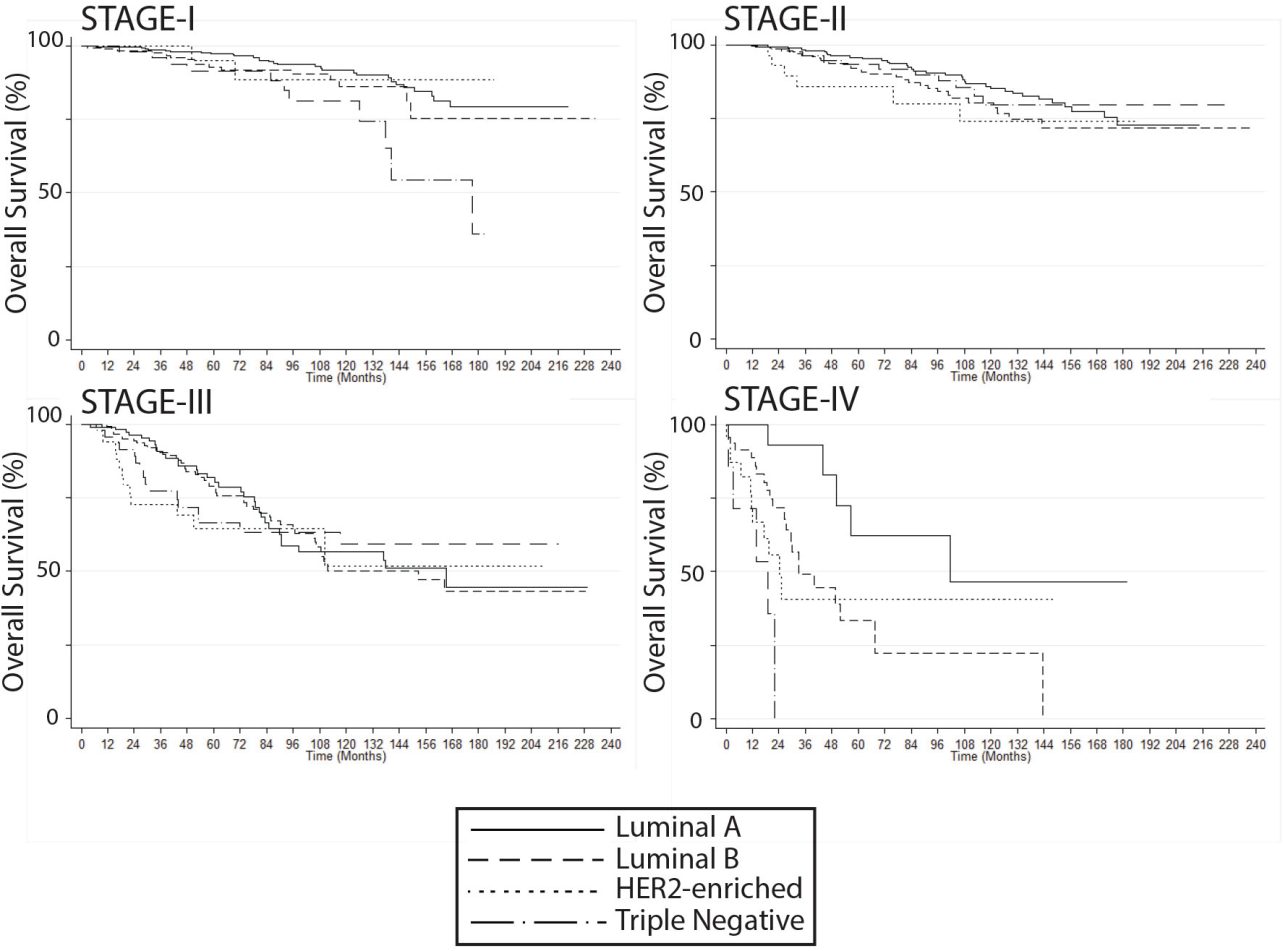
Breast cancer overall survival by molecular subtype, stratified by stage at diagnosis (Kaplan Meier).

Median follow-up was 57.5 months (0–237), with 451 deaths (16.6%). Five-year overall survival (OS) for the entire group was 88.6%. OS at 5 years was statistically similar between the first and second decade of the study (1997–2007: 88.8% versus 2008–2018: 88.3%, *p* = 0,621). As expected, survival rates were significantly lower on stage IV cases (Figure 1C). Conversely, patients diagnosed by mammography had significantly better survival rates versus symptoms: 5-year survival was 96% versus 86.1%, respectively, and 10-year survival was 90.1% versus 72.4%, respectively (*p* < 0.001; Figure 1D). Five-year, 10-year and 15-year OS values by stage or tumour subtype are summarised in Supplementary Table S1. In addition, OS rates by BC subtype, Luminal versus non-luminal, by HER2 status, by stage or HG are shown in Supplementary Figures S1 and S2.

## Multivariate analysis

Supplementary Table S2 shows the results of the multivariate analysis for OS. Period of study was not included in this analysis since it showed no differences between the 2 compared decades. Since HG is included into our definition of BC subtype it was also excluded from analysis. Age, RAC, stage and BC subtype were associated to OS. As expected, stage III/IV mortality rates were significantly higher than stage I. In addition, mortality rates of HER2-enriched were 1.98-fold higher versus luminal-A. Finally, symptomatic patients had a 1.77-fold higher estimated mortality against mammography. Supplementary Table S3 compares clinicopathological variables between patients consulting for altered mammograms and those with signs or symptoms of BC.

## Discussion

Since their introduction in the early 1980s the widespread use of mammography screenings have demonstrated a concomitant (and expected) increase in early-stage BC incidence. This has been confirmed by demographic data from the Surveillance, Epidemiology and End Results (SEER) database in the United States (US), over the 1973–2008 period [2]. More recently, a study by Verdial *et al* [8] also analysed the SEER database over the 1973–2013 period and further confirmed these findings, but also showed a decline in overall BC incidence in the 1998–2003 period followed by stabilisation in the ‘post-2003’-era. This study also shows that the increase in BC incidence is observed among women aged 50–80 years and establishes that median age for BC diagnosis has risen to 61 years in the US. Accordingly, our study found a median age at diagnosis *n* = 55 years that remained unchanged over the 1997–2018 period. However, we demonstrate no changes in early stage BC diagnosis and a surprising increase in stage IV BC cases over the assessed period (Figure 1A and 1B). A couple of reasons could explain these discrepancies: first, our study was performed in a private university hospital where early detection strategies were implemented in the early 1990s, therefore were probably observed prior to the time period covered by our study. In addition, attended patients can afford or have better access to preventive screenings. Second, the increase in stage IV cases could be attributed to better stratification derived from improved more sensitive imaging techniques.

## Conclusions

Previous studies demonstrate that patients that come to the clinic after the appearance of signs/symptoms (versus mammography) almost double their risk of death. Conversely, breast neoplasms diagnosed by mammography display better prognosis, survival rates [9] and reduced mortality [10]. Our study confirms these findings and supports the efficacy of diagnostic screening by mammography in order to reduce BC mortality.

## Ethics approval

The Scientific and Ethics committee at the Pontificia Universidad Catolica de Chile approved this research. Given the retrospective nature of the study no sensitive information was obtained from patients and all data were anonymised.

## Conflict of interest

The authors declare no conflicts of interest.

## Funding statement

FONDECYT-Iniciacion grant #11161103 (CS).

## Figures and Tables

**Table 1.** Clinical characteristics of breast cancer patients diagnosed in the 1997–2018 period.

Age at diagnosis, average ± SD	55.7 ± 13.13 years
**Tumour stage at diagnosis**	**N, (%)**
Stage I	878 (35.5%)
Stage II	961 (38.9%)
Stage III	496 (20.1%)
Stage IV	135 (5.5%)
**BC tumour subtype**	**N, (%)**
Luminal A	1,083 (47.4%)
Luminal B	776 (33.9%)
HER2-enriched	160 (7.0%)
Triple Negative	267 (11.7%)

**Table 2.** Tumour stage at diagnosis by reason for attending the clinic.

Tumour stage	Mammography %	Symptoms %
Stage I	58.5%	17.45%
Stage II	31.7%	45.3%
Stage III	7.90	29.7
Stage IV	1.77	7.52%

**Table S1.** Five, ten and fifteen year overall survival (OS) by tumor stage or BC subtype.

	5-year OS	10-year OS	15-year OS
**Tumor stage**	**% (*n*)**	**% (*n*)**	**% (*n*)**
Stage I (*n* = 878)	96.2 (476)	89.6 (228)	77.6 (49)
Stage II (*n* = 961)	93.4 (480)	80.3 (249)	71.4 (53)
Stage III (*n* = 496)	75.7 (198)	51.1 (84)	41.1 (17)
Stage IV (*n* = 135)	40 (18)	24.4 (4)	-
**BC subtype**	**% (*n*)**	**% (*n*)**	**% (*n*)**
Luminal A (*n* = 1,083)	93.8 (515)	83.7 (25)	70.5 (46)
Luminal B (*n* = 456)	86.5 (206)	68.3 (57)	58.7 (6)
HER2-enriched (*n* = 160)	76.8 (57)	63.6 (18)	63.6 (3)
Triple Negative (*n* = 271)	85.2 (124)	73.7 (49)	58.6 (7)

**Table S2.** Multivariate analysis of survival: Cox Regression.

	Hazard Ratio	*p*-value	CI: 95%	
Age	1.021	0.001	1.009	1.033
Stage I	(ref.)	-	-	-
Stage II	0.958	0.868	0.580	1.584
Stage III	2.759	0.000	1.689	4.507
Stage IV	13.017	0.000	7.139	23.734
Luminal A	(ref.)	-	-	-
Luminal B	1.338	0.143	0.906	1.977
HER2 enriched	1.978	0.040	1.031	3.795
Triple negative	1.460	0.134	0.890	2.395
RAC* Symptoms	1.765	0.011	1.1408	2.7305

Abbreviations: RAC= Reason for Attending the Clinic, CI 95%= Confidence Interval 95%

Ref.: this value was used as reference

**Table S3.** Comparison of clinicopathological variables between patients consulting for altered mammograms and those with signs or symptoms of BC.

N: 1,754 patients	Mammograms (N: 648)	Signs or symptoms (N: 1,106)	p-value
Age at diagnosis, average ± SD	56.5 ± 10.6 years	54.5 ± 14.8 years	0.002
Tumor stage at diagnosis	N, (%)		
Stage I	363 (58.5%)	181 (17.5%)	<0.0001
Stage II	197 (31.8%)	470 (45.3%)	
Stage III	49 (7.9%)	308 (29.7%)	
Stage IV	11 (1.8%)	78 (7.5%)	
BC tumor subtype	N, (%)		
Luminal A	355 (62.9%)	368 (38.6%)	<0.0001
Luminal B	133 (23.6%)	350 (36.7%)	
HER2-enriched	27 (4.8%)	73 (7.7%)	
Triple Negative	49 (8.7%)	163 (17.1%)	
